# Digitally-defined ultrathin transparent wireless sensor network for room-scale imperceptible ambient intelligence

**DOI:** 10.1038/s41528-024-00293-4

**Published:** 2024-02-06

**Authors:** Yunxia Jin, Mengxia Yu, Dat T. Nguyen, Xin Yang, Zhipeng Li, Ze Xiong, Chenhui Li, Yuxin Liu, Yong Lin Kong, John S. Ho

**Affiliations:** 1 Institute for Health Innovation and Technology, National University of Singapore, Singapore 117599, Singapore.; 2 Department of Biomedical Engineering, National University of Singapore, Singapore 119276, Singapore.; 3 Department of Electrical and Computer Engineering, National University of Singapore, Singapore 117583, Singapore.; 4 Integrative Sciences and Engineering Programme, National University of Singapore Graduate School, Singapore 119077, Singapore.; 5 The N.1 Institute for Health, National University of Singapore, Singapore 117456, Singapore.; 6 Institute of Materials Research and Engineering, Agency for Science Technology and Research, Singapore 138634, Singapore.; 7 Department of Mechanical Engineering, University of Utah, Salt Lake City, UT 84112, USA.

## Abstract

Wireless and battery-free radio-frequency (RF) sensors can be used to create physical spaces that ambiently sense and respond to human activities. Making such sensors ultra-flexible and transparent is important to preserve the aesthetics of living environments, accommodate daily activities, and functionally integrate with objects. However, existing RF sensors are unable to simultaneously achieve high transparency, flexibility, and the electrical conductivity required for remote room-scale operation. Here, we report 4.5 μm RF tag sensors achieving transparency exceeding 90% that provide capabilities in room-scale ambient wireless sensing. We develop a laser-assisted water-based adhesion-reversion process to digitally realize computer-aided RF design at scale. By individually tagging multiple objects and regions of the human body, we demonstrate multiplexed wireless tracking of human-environment interactions and physiological signals at a range of up to 8 m. These radio-frequency identification sensors open opportunities for non-intrusive wireless sensing of daily living spaces for applications in health monitoring and elderly care.

## INTRODUCTION

Ambient intelligence promises a future of sensor-embedded physical environments that are sensitive and responsive to human activities^1-3^. Particularly, the ability to incorporate intelligence into an otherwise passive surrounding environment is of significant interest in healthcare. Such technology could be used to collect longitudinal data, gain insight into a person’s health, and enable remote patient monitoring. Of the various types of sensors used for ambient intelligence, ultra-high radio-frequency identification (UHF-RFID) is particularly suited for the tracking of health-critical behaviors because of its long readout range (up to approximately 10 m), robustness to occlusions, and enhanced privacy compared to optical or acoustic sensors^4-10^. Compared with near-field approaches that have significant restrictions on user mobility due to the limited power transfer range and sensitivity to alignment, the RFID technology allows remote sensing at the range of meters simultaneously from multiple spatially distributed objects^5,6^.

Optical and mechanical imperceptibility is crucial for unobtrusive wireless sensing and ambient intelligence^11-16^ to accommodate users’ regular daily activities without interference. However, existing UHF-RFID devices cannot achieve the transparency and flexibility that is required to create far-field imperceptible wireless interfaces^14-25^. High-aspect ratio conductive nanomaterials, such as metallic nanowires, can achieve comparable conductivity with transparent conductive oxides (such as ITO) at RF frequencies and are mechanically flexible^26-30^. Current fabrication processes for such nanowire networks, however, rely either on printing strategies, which suffer from poor conductivity of a figure of merit σ_DC_/σ_Op_ below 100 that is only ~25% of the film prepared with rod-coating or limited resolution because of the inherent instability of evaporative-driven assembly of a low-viscosity ink^31-38^, or laser ablation, which results in thermal damage and residue that compromises optical tranparency^39-42^. As a result, the working range of existing transparent RF sensors is limited to centimeter scale and room-scale operation has been achieved only by opaque devices.

**Fig. 1:**
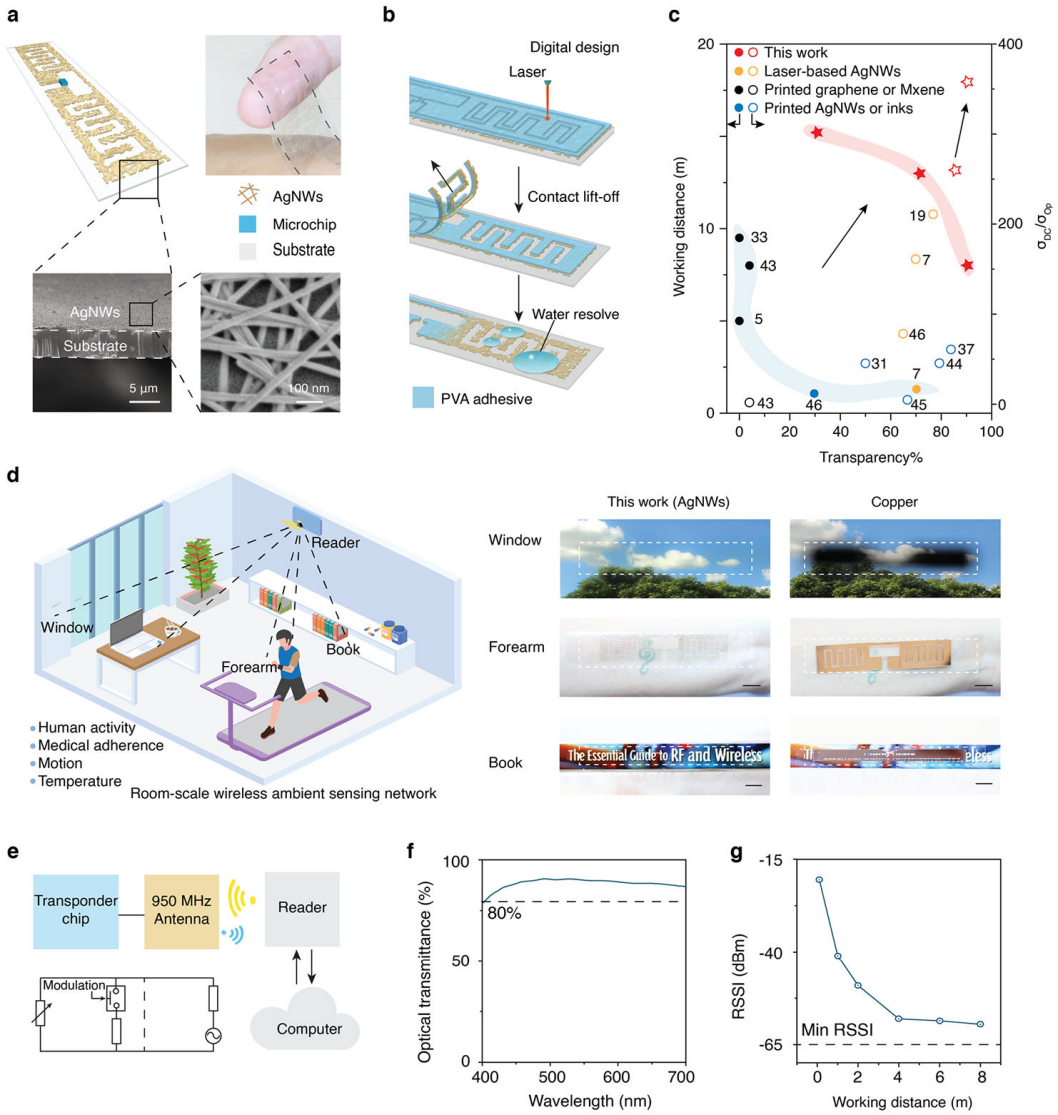
Ambient wireless sensing with ultrathin transparent RF sensors. **a** Ultrathin and transparent RF sensor based on AgNWs. Inset shows SEM image of the cross-section and top surface. **b** Schematic of the fabrication process. The antenna pattern is created by laser cutting PVA adhesive placed on the AgNW network following the digital design in computer, removing extraneous AgNW via contact lift-off of PVA adhesive, and exposing the remaining AgNWs area to form pattern via water resolving the adhesion from PVA adhesive in washing. **c** Working distance and conductivity (figure of merit, σ_DC_/σ_Op_) of patterns in this work compared with that of other reported fabrication methods. The literature citation numbers are adjacent to the dots or circles. **d** Illustration of ambient wireless temperature monitoring, activity tracking, and usage detection from a network of RF tag sensors through an RFID reader. Bottom are images of the transparent RF tag sensors placed on a window, left forearm, and a book compared to tag sensors fabricated from copper. **e** Block diagram shows the device working principle. **f** Optical transmittance of 4.5 μm-thick tag sensor against a blank PET substrate. **g** Operating range of the tag sensor in an indoor environment at a transmit power of 36 dBm.

To address the challenges, we developed ultrathin and transparent UHF-RFID sensors made from silver nanowire (AgNW) networks that provide capabilities in room-scale wireless ambient sensing (Fig. 1a-c, Supplementary Table 1). We create these sensors using a newly developed adhesion-reversion-driven contact lift-off technique integrated with facile laser cutting technology to directly pattern AgNW networks on ultrathin (< 4.5 μm) polymer substrates without altering the optical and electrical properties. We fabricate RF sensors that achieve high transparency (> 90% across the visible spectrum) and mechanical flexibility while enabling UHF-RFID operation at a range of up to 8 m. We demonstrate the utility of these sensors for ambient intelligence by attaching the sensors conformally on three-dimensional objects and human skin for multiplexed wireless tracking of object temperature, human activities, and respiration signals from different locations using a single wall-mounted RF reader.

## RESULTS

### Transparent ambient wireless sensor

Figure 1 shows the overview of the room-scale ambient wireless sensing system using optically transparent and ultrathin RF tag sensors. Owing to the ultrathin and transparent form factor, the tag sensors can be conformally attached onto three-dimensional objects for applications such as temperature monitoring, object usage detection, and human activity tracking (Fig. 1b-d). Figure 1b shows the fabrication process. The polyvinyl alcohol (PVA) tape (50 μm thickness) is attached on AgNW-coated thin film, and cut using a low-cost CO_2_ laser following the computer-controlled digital design without penetrating the substrate. AgNWs are then removed from the extraneous regions by contact lift-off of the PVA adhesive. The desired pattern is finally obtained by washing away the remaining PVA tape with water to expose the underlying AgNWs. The fabrication method enables computer-aided RF design digitally patterned at scale using conductive AgNW networks on ultrathin substrates through laser defined adhesion reversion of water-soluble PVA tape to AgNWs. Since this process does not require high-temperature annealing, chemical etching or transfer to another substrate, the patterns have optical and electrical properties that are nearly identical to the pristine network (Supplementary Figs. 1, 2), in which the sheet resistance fluctuations are less than 10% and transmittances are almost the same. As a result, it shows over three times higher in conductivity compared to printed patterns and one more time to other laser-based patterns at high transparency of 90%, exhibiting significantly improved working distance from centimeter to meters scale at high transparency (Fig. 1c)^5,7,19,31-33,37,36,42-45^. Images of tag sensors attached to a window, placed on the left forearm, and on the spine of a book illustrate the optical transparency and mechanical flexibility of the tag sensors compared to copper tag sensors based on the same antenna designs (Fig. 1d, e, Supplementary Figs. 1-3). Specifically, when applied to a network on a 4.5 μm polyethylene terephthalate (PET) substrate, the process yields antenna with optical transmittance around 90% at 550 nm (Fig. 1f) and sheet resistance of 9.7 Ω sq^−1^. An RF tag sensor created by attaching a 0.5 mm × 0.5 mm × 0.1 mm microchip to the antenna achieves an operating range of up to 8 m from a commercial RFID reader at an output power of 36 dBm (Fig. 1g, Methods). By increasing the density of the AgNW network, the range can extend to 15.2 m with RF tag sensor of 1.7 Ω sq^−1^, which is within 84.4% of the operating range of tag sensors based on non-transparent copper.

**Fig. 2:**
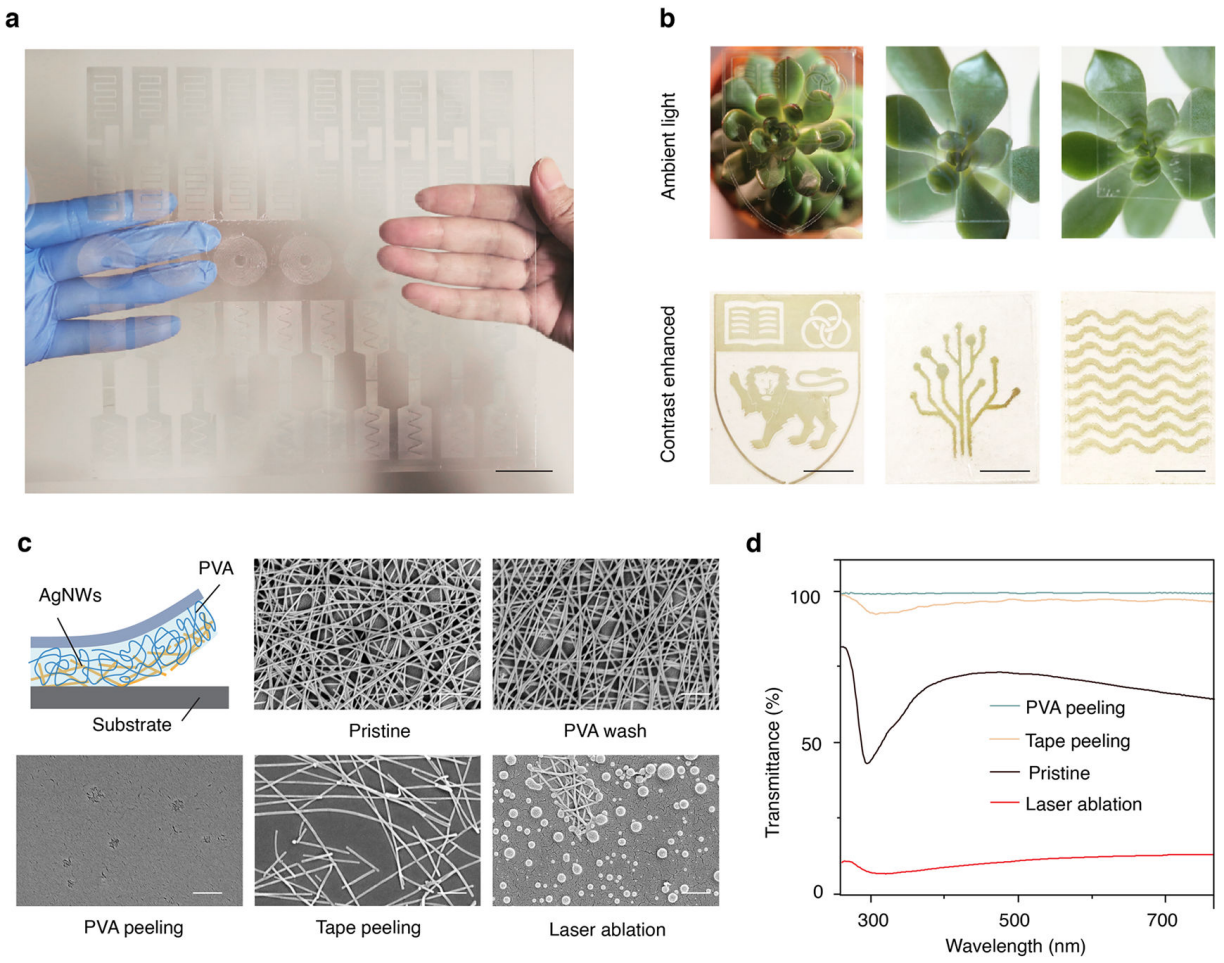
Fabrication characteristics. **a** Image of 27 tag sensors produced on a 20 cm × 18 cm substrate using the fabrication method. Scale bar, 3 cm. **b** Images of fabricated patterns under ambient lighting conditions and on a uniform white background with digital contrast enhancement. Scale bar is 3 mm. **c** Top row shows schematic of peeling step of PVA adhesive and SEM images of the pristine AgNWs network and the network after washing away the PVA adhesive. Bottom row shows regions where the network is removed by peeling PVA adhesive, peeling with tape, and laser ablation. Scale bar is 500 nm. **d** Optical transmittance of the pristine AgNW network and regions where the network is removed by PVA peeling, tape peeling and laser ablation against a blank PET substrate.

Figure 2a and Supplementary Fig. 4 show scalable manufacturing of 27 tag sensors on a 20 cm × 18 cm sheet. We also demonstrated the ability to create complex patterns, including a logo and meandered lines, with feature sizes that are visually almost invisible under ambient lighting conditions from AgNWs network with sheet resistance of 6.5 Ω sq^−1^, requiring digital contrast enhancement on a visually uniform background to reveal the patterns (Fig. 2b). To show how our process enables these properties, we compared three removal strategies in worse case of higher AgNWs deposition density at 71.4% of transmittance which is more difficult to be removed via one-pass peeling: (i) peeling with PVA adhesive in air, (ii) peeling with tape, and (iii) etching with laser ablation (see Methods). Scanning electron microscopy (SEM) images (Fig. 2c) show that our technique successfully removes the AgNWs completely from the substrate, resulting in a near 100% optical transmittance against the blank substrate and no electrical conductivity (Fig. 2d, Supplementary Fig. 5). Supplementary Fig. 6 shows a clear AgNWs edge after removing part of AgNWs using our technique where one side covered by PVA adhesive is completely removed with peeling while the other side remains intact. In contrast, the tape leaves a thin layer of residual AgNWs with an optical transmittance of 97% and sheet resistance of 122.3 Ω sq^−1^. We attribute this to the high mechanical compliance of PVA adhesive that enables a conformal adherence with AgNWs networks. The laser ablation appears to be effective in disrupting the conductive network, while Ag nanoparticles residues resulting from high-energy irradiation melting (Fig. 2c) causes a reduction of optical transparency to 12% (Fig. 2d, Supplementary Fig. 7). Further, in contrast to our approach, the serial process of laser ablation (10% of laser cutting speed) is not suitable for large-scale production. We observed no change in the AgNWs network morphology and density before and after removing the PVA adhesive with water (< 1 min) (Fig. 2c). The fabrication method yielded similar results when applied to a hybrid network of AgNWs and graphene oxide (GO) (Supplementary Fig. 8), which is widely used to improve the conductivity and stability of nanowire networks^46^, as well as to a variety of substrates, including rigid glass, 100 μm-thick PET, and 4.5 μm-thick PET (Supplementary Fig. 9).

### Antenna performance

**Fig. 3:**
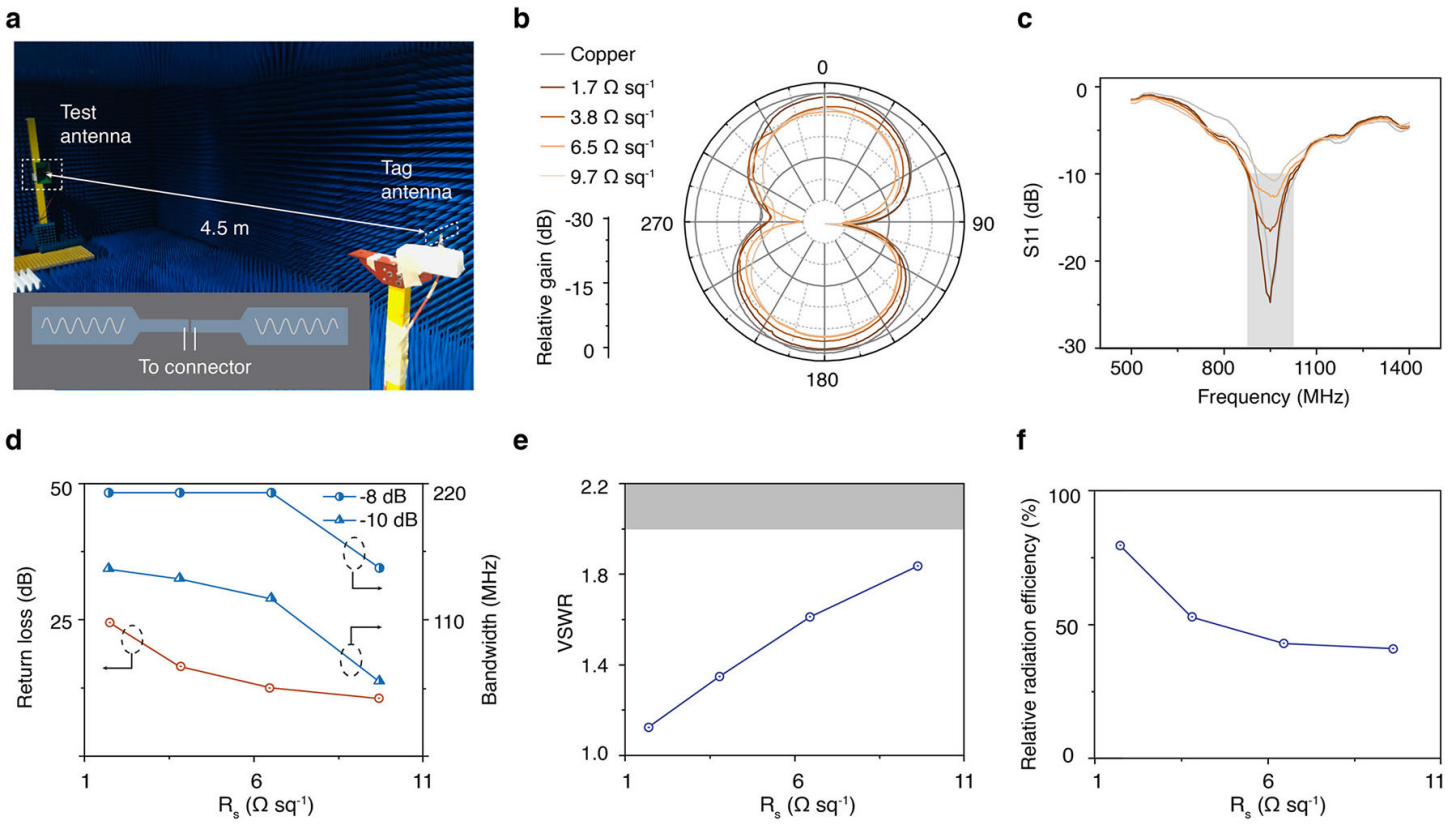
Transparent antenna performance. **a** Image of the experimental setup in an anechoic chamber. Inset shows connector placement. **b** Measured radiation patterns (E-plane) from antennas fabricated from transparent AgNW networks with varying sheet resistances relative to the gain of a copper antenna. **c** Reflection coefficient S11 spectra of the antennas. **d** Input return loss, 8 dB bandwidth, and 10 dB bandwidth as a function of the sheet resistance R_s_. **e** VSWR as a function of R_s_. **f** Relative radiation efficiency as a function of R_s_ compared to the copper antenna.

We next studied the performance of RF antennas produced using our method by fabricating a 950 MHz meandered dipole antenna from AgNW networks with varying degrees of optical transparency and electrical conductivity (Supplementary Fig. 10). To ensure the conductivity measurement accuracy, we repeat on sample with 5 different locations (Supplementary Fig. 11). Figure 3a shows the characterization setup in which the antennas are attached to a SMA-type connector via silver paint and placed in an anechoic chamber (see Methods). Transparent antennas with sheet resistances ranging from 1.7 to 9.7 Ω sq^−1^ produced a dipole radiation pattern that closely matches that of the same antenna fabricated from copper (Fig. 3b). Owing to the increased resistive loss of the antenna, the gain of the antenna decreases with the optical transparency. For the antenna fabricated from the 9.7 Ω sq^−1^ network (~90% transparency at 550 nm), the gain is −3.8 dB of the copper antenna.

We also characterized the resonances of the antennas around the operating frequency of 950 MHz by measuring their scattering parameters S_11_. Figure 3c shows that position of the resonant dips in S_11_ spectra of the transparent antennas are closely aligned with that of the copper antenna, which indicates that the fabrication method has sufficiently high resolution to accurately produce electromagnetic structures in this frequency range. The return loss is greater than 10 dB for all of the antennas, and is the lowest for the antenna fabricated from the 9.7 Ω sq^−1^ network with highest transparency (Fig. 3d). The operating bandwidth exceeds 210 MHz (10 dB criteria) or 150 MHz (8 dB criteria) for antennas fabricated from networks with sheet resistance lower than 6.5 Ω sq^−1^, meeting requirements of standard RFID protocols even at the highest transparency based on the 9.7 Ω sq^−1^ network^47,48^. In all cases, the voltage standing wave ratio (VSWR) was less than 2, indicating that the antennas are impedance-matched to a standard 50 Ω port. Measurements using a test antenna placed at a distance of 4.5 m (see Methods) show that the radiation efficiency of the transparent antennas are within 40 to 80% of the copper antenna.

### RF tag sensor performance

**Fig. 4:**
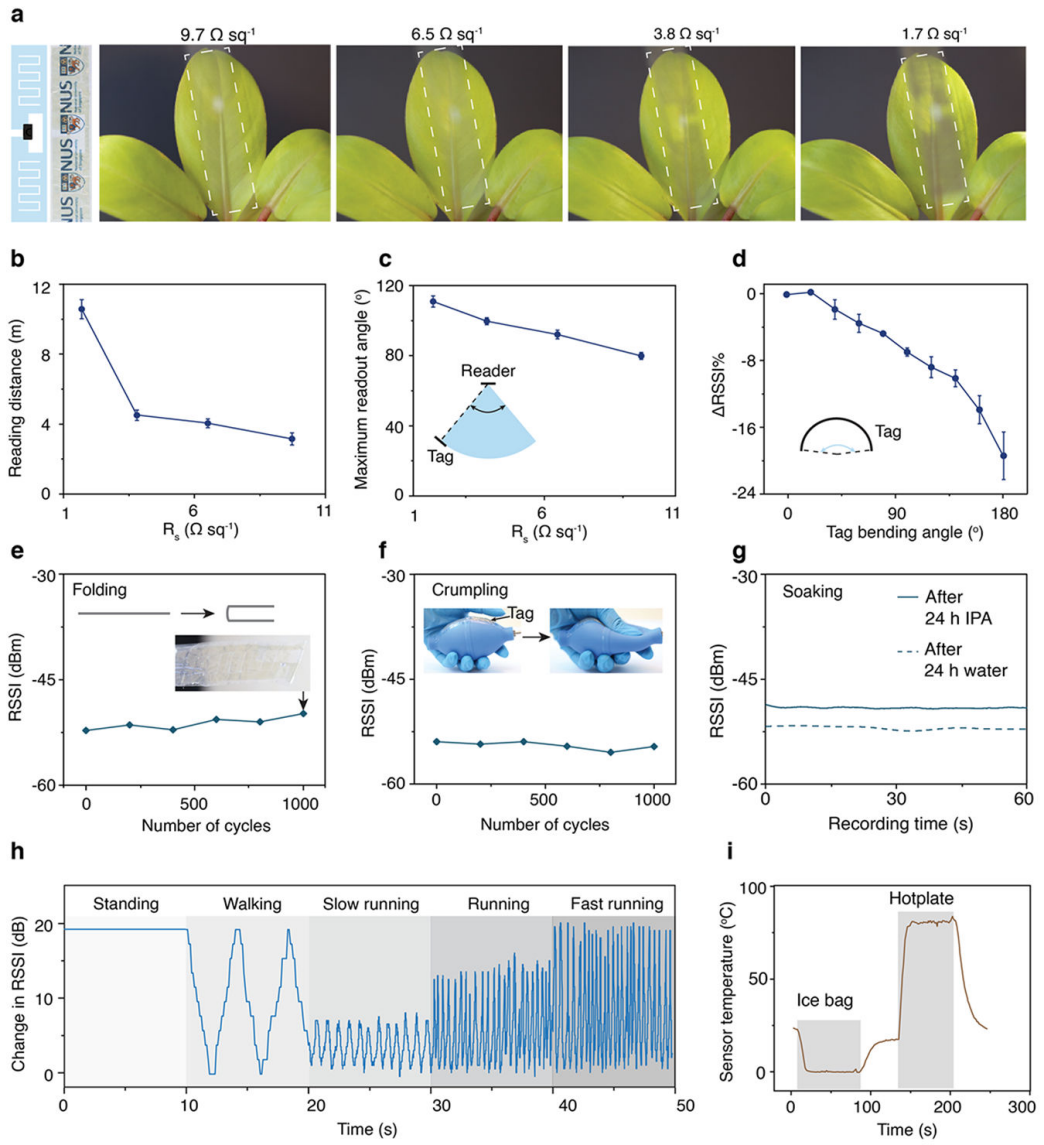
RF tag sensor performance. **a** Images of 4.5 μm-thick transparent tag sensors fabricated from AgNW networks with varying sheet resistance under ambient lighting conditions. The design and the photograph of the antenna is shown on the left. **b** Operating range of the transparent tag sensors using a reader antenna with gain of 6 dBi and maximum transmit power of 30 dBm. Error bar is the standard deviation of the operating range. **c** Maximum angle of interrogation of the RF tag sensors. The tag sensors are placed 2 m from a reader antenna with a gain of 6 dBi and a maximum transmit power of 30 dBm. Error bar is the standard deviation of the maximum angle. **d** Change in RSSI as a function of bending angle of the tag sensor. Measurements are performed using a tag sensor fabricated from a 9.7 Ω sq^−1^ network (90% transparency) at 2 m range from the reader. Error bar is the standard deviation of the change of RSSI. **e–g** RSSI over 1000 cycles of folding (**e**), 1000 cycles of crumpling (**f**), and after soaking for 24 h in water and IPA (**g**) from a tag sensor with 9.7 Ω sq^−1^ network (90% transparency) at 2 m range from the reader. **h** Change in RSSI obtained from a tag sensor attached onto the forearm of a human subject during physical activity. **i** Wireless temperature readout from the tag sensor when it is placed on an ice bag and a hotplate.

To evaluate the system characteristics, we attached transponder chips to the transparent antennas and performed a variety of wireless sensing tasks using a commercially available RFID reader (see Methods). Figure 4a shows images of four meandered tag sensors fabricated from AgNW networks with varying sheet resistances and optical transparencies under ambient lighting conditions. The reader uses a linearly polarized antenna with a gain of 6 dBi and a maximum output power of 30 dBm in compliance with regulatory requirements for electromagnetic emission in uncontrolled environments (see Methods)^49,50^. Using this configuration, wireless sensing is primarily limited by the amount of power extracted by the tag sensor antenna, which must exceed the sensitivity of the transponder chip (about −20 dBm) in order for the readout to be successful. The transparent meandered tag sensor fabricated from 9.7 Ω sq^−1^ AgNW networks with 90% transparency has a maximum readout range of 3.0 m at normal incidence from the reader antenna. The range can be extended up to 10.6 m using tag sensors based on the denser 1.7 Ω sq^−1^ network, which is within 81.5% of readout range achieved with the tag sensor fabricated using copper (Fig. 4b). Experiments with alternative tag sensor designs yielded similar readout ranges (Supplementary Figs. 12, 13 and Supplementary Movie 1) and performance relative to their counterparts based on copper. We also tested the angular coverage of wireless sensing by varying the angle of incidence between the reader and the tag sensor. The maximum angle of incidence decreases from 55° to 40° as the sheet resistance of the AgNW network increases from 1.7 to 9.7 Ω sq^−1^, compared to 70° for the copper-based tag sensor (Fig. 4c). We then studied the tag detection under shape deformation by bending the tag sensor. We note that even at 180° bending, the tag sensor remains functional with only 22% decrease in Received Signal Strength Indicator (RSSI) (Fig. 4d, Supplementary Fig. 14).

We further characterized the robustness of the fabricated tag sensors to mechanical stresses and wetting. Repeated folding and crumpling of the tag sensor fabricated from the 9.7 Ω sq^−1^ network, which exhibits the highest transparency, demonstrate no significant change in the fidelity of wireless readout, as measured by the RSSI (Fig. 4e, f, Supplementary Movie 2). In contrast, tag sensors fabricated from copper failed to recover to their original shape after folding, leading to reduction of wireless readout ability (Supplementary Fig. 15). Signals obtained from the tag sensors were stable following 24 h of soaking in isopropyl alcohol (IPA) and water even without encapsulating the AgNW network in a protective material (Fig. 4g). The mechanical characteristics resulting from the ultrathin profile of the tag sensors enable them to be conformally attached on the human body for sensing human activity. Figure 4h shows an example in which attaching a transparent tag sensor to the forearm of a human subject allows tracking of standing, walking, and running at various speeds through the RSSI of the signal acquired by the reader. In addition, the tag sensors can be separately configured with sensors whose data can be transmitted to the reader during the readout process. For instance, transparent tag sensors configured with thermal sensors can transmit environmental temperature between 0 and 85 °C with high accuracy compared to a thermocouple (Fig. 4i, Supplementary Fig. 16).

### Imperceptible wireless sensor network

**Fig. 5:**
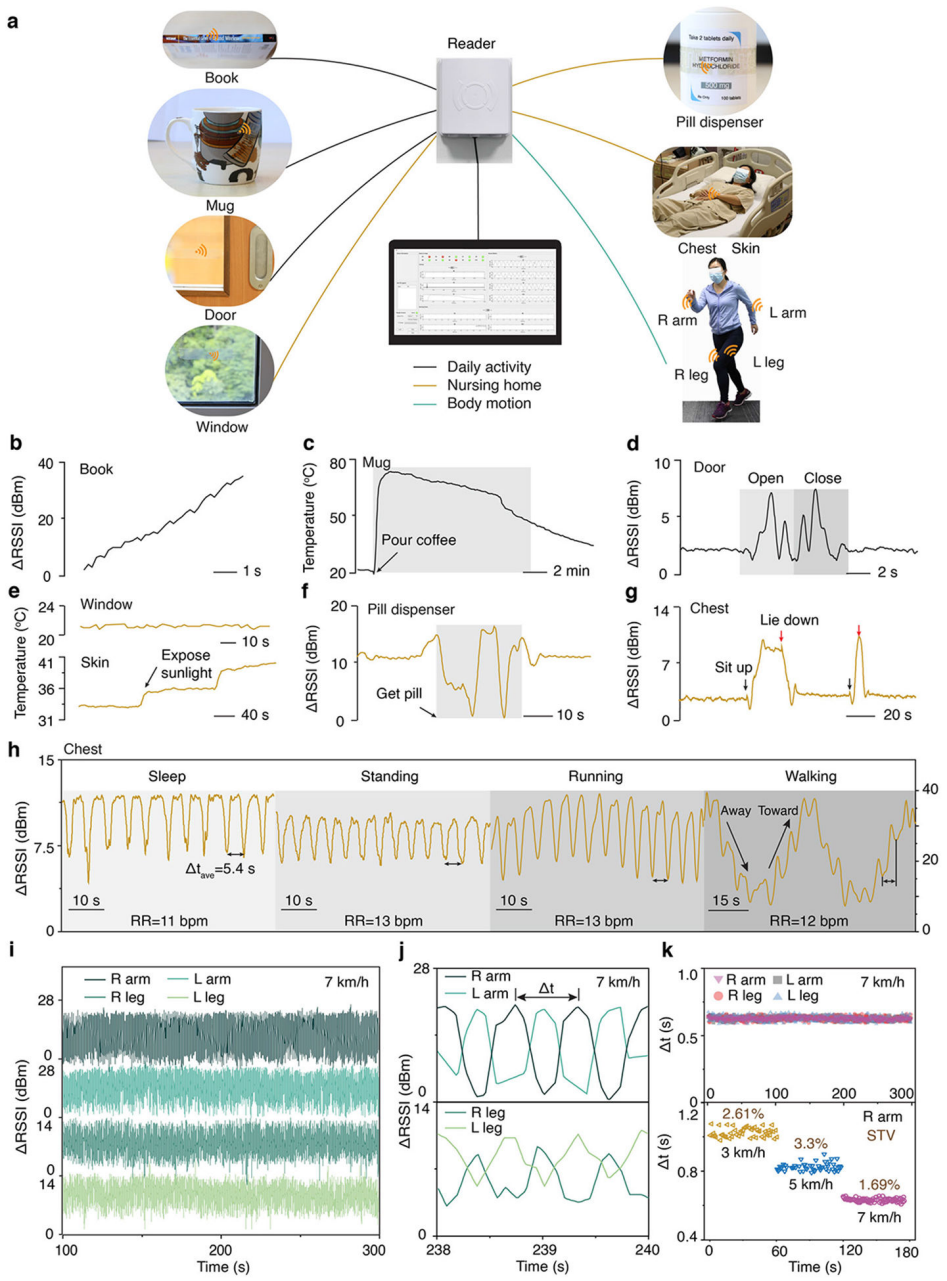
Room-scale ambient wireless sensing. **a** Images of transparent tag sensors with 4.5 μm thickness placed on various objects for multiplexed activity tracking. A RFID reader is placed on the wall in a room and streams data to a graphical user interface. **b–d** Multiplexed RSSI and temperature data when the subject moves a book through the room (**b**), pours hot coffee into a mug (**c**), and opens and closes the door (**d**). **e–g** Multiplexed RSSI and temperature data when the subject exposes her skin to sunlight (**e**), obtains medicine from a pill dispenser (**f**), and sits upright and lies down on a bed (**g**). **h** RSSI obtained from a tag sensor placed on the chest of a subject during sleeping, standing, running, and walking. The peak-to-peak interval indicates the respiration rate (RR) of the subject. **i** RSSI obtained from 4 tag sensors placed on the arms and legs of a subject jogging on a treadmill at a speed of 7 km h^−1^. **j** Exploded view of the RSSI trace showing the stride time Δ*t* given by the peak-to-peak interval. **k** Stride time Δ*t* obtained from arms and legs when the speed is held constant at 7 km h^−1^ (top) and from right arm but changing the speed from 3 to 7 km h^−1^ (bottom). STV, stride time variability.

We demonstrated the utility of the fabricated tag sensors for ambient wireless sensing by deploying multiple tag sensors in an indoor environment for multiplexed detection of various human activities. A RFID reader was mounted on a wall and connected to a graphical user interface that displays the RSSI obtained from each tag sensor and, if applicable, data from the integrated temperature sensor. Capabilities were evaluated in three setups: (i) multiple object tracking using tag sensors placed on the spine of a book, on the exterior of a mug, and on a door; (ii) ambient temperature and human activity monitoring using tag sensors placed on a window, on a pill dispenser, and on the chest; (iii) gait detection using tag sensors placed on the left and right arms and legs (Fig. 5a, Supplementary Movie 3). In the first setup, the RSSI trace and the temperature data revealed when the book is moved through the room, hot coffee is poured in the mug, and the door is opened and closed (Fig. 5b-d). These activities were individually identifiable because of the unique ID associated with each tag sensor and multiplexing protocols used by the reader. In the second setup, a tag sensor placed on the window tracked the room temperature (about 21 °C) while a tag sensor attached to the forearm captured a ~ 3 °C increase in skin temperature when exposed to elevated amounts of sunlight. At the same time, RSSI traces obtained from tag sensors on the pill dispenser and on the chest of an occupant showed variations of ± 10 dB when the subject opened the pill dispenser and changed from an upright to lying down position (Fig. 5e-g). Further experiments showed that RSSI trace obtained from the tag sensor attached to the chest exhibits periodic variations whose peak-to-peak interval can be used to measure the respiration rate of the subject, as shown during sleeping, standing, walking, and running (Fig. 5h). Finally, in the third setup, four tag sensors placed on the arms and legs of the subject produced independent RSSI traces (Fig. 5i, Supplementary Fig. 17, and Movie 4) whose peak-to-peak intervals corresponded to the stride time Δ*t* of a subject on a treadmill (Fig. 5j). The data revealed that Δ*t* obtained from each tag sensor were not significantly different, which is consistent with the motion of a healthy subject. Increasing the speed of the treadmill from 3 to 7 km h^−1^ resulted in a corresponding decrease in the stride time from 1.03 to 0.64 s, indicating an increased gait. Analysis of the stride time variability (STV) – an important physiological indicator of fatigue – showed that the STV varies from 2.36% to 3.06% depending on the gait, which is consistent with the 3% benchmark for healthy subjects with normal gait control^51,52^.

## DISCUSSION

This paper demonstrates ultrathin and transparent RF tag sensors that provide capabilities in imperceptible room-scale ambient wireless sensing. We create these tag sensors using a facile scalable fabrication method that combines laser cutting technology to digitally define the pattern and an adhesion reversion process to directly expose the pattern of transparent conductive AgNW networks on ultrathin substrates. Characterization experiments show that the tag sensors achieve thickness less than 4.5 μm, transparency exceeding 90% across the visible spectrum, and RF characteristics compatible with room-scale UHF-RFID wireless operation. Multiplexed operation of multiple tag sensors attached to objects and the human body in complex indoor environments using a UHF-RFID reader demonstrate the utility of the tag sensors for continuously tracking health-relevant behaviors, such as interactions with objects, temperature, and human body motions in daily living spaces.

Ultrathin and transparent RF tag sensors may open a range of opportunities to gain insight about human health through ambient intelligence. In daily living spaces, RF tag sensors could be used to track hand hygiene, medication adherence, and physical activity and provide feedback to promote healthy behaviors^53,54^. They can also be used in elderly homes to detect falls and impairment of one’s ability to perform activities of daily life, such as dressing, bathing, and eating^55,56^. In these contexts, the mechanical and optical imperceptibility of the tag sensors may reduce the obtrusiveness of the technology and increase user acceptance, while protecting privacy by only allowing labeled objects to be tracked within a defined region. To make sense of the datasets provided by these tag sensors, machine learning models that are computationally efficient, privacy-preserving, and capable of detecting rare and infrequent events will need to be developed^57,58^. Furthermore, inferring motions using machine learning could provide initial risk assessment before clinical diagnosis^59,60^.

With further development, our fabrication process may be adapted for large-scale manufacturing of RF tag sensors for practical widespread use. The key patterning step relies on laser cutting which is already well-established for RFID and various devices manufacturing at large volumes^61-65^. In this regard, methods to increase the throughput of laser cutting based on multi-optics systems could be applied here^66^. Automation of the peeling step can be achieved with roll-to-roll processes, such as by using tension rollers^67^. Furthermore, the process could be extended to the fabrication of transparent RF devices that demand higher resolution, such as those operating at frequencies above 1 GHz, by using an ultraviolet laser cutter with a spot size of 10–20 μm^68^. Our process could also be applied to pattern other classes of nanowire structures and functional materials, such as carbon nanotubes, to incorporate additional sensing functionalities in the RF tag sensors^69^. Further, the conductivities can be improved by increasing the aspect ratio of AgNW^70^ or introducing composites^70^, which have the potential to yield increased RF performance.

## METHODS

### Fabrication process

The AgNW dispersion (Zhejiang Kechuang Advanced Materials Co., Ltd, 10 mg ml^−1^, average diameter ~30 nm and length ~15 μm) was diluted to 1 mg ml^−1^ with isopropyl alcohol. The 4.5 μm (Sigma Aldrich) and 100 μm (Dupont Teijin Films) PET substrates were cleaned with deionized water, alcohol, and acetone, and then dried in the oven. The diluted AgNW dispersion was then coated onto the PET substrate using a Meyer rod (size 6). The conductivity and transparency of the resulting AgNWs film was tuned by changing the coating times, which varied density of the AgNW network deposited on the substrate. Water-soluble tape (3 M 5414, PVA adhesive, 50 μm thickness) was attached onto the surface of AgNW/PET film and pressed to establish a seamless contact. The tape was cut into the desired pattern using a laser cutter (Universal Laser Systems, VLS 2.30). The parameters of the laser cutter (0.4% power, 3% speed, 1000 pixels-per-inch, 5 image density, vector mode) were set to cut through the tape but not the substrate. The tape in the extraneous areas was manually peeled, leaving only the desired pattern on the substrate. The remaining tape was removed by soaking in water for 5 min. To improve the adhesion of AgNWs to substrate PET and wettability of AgNWs dispersion during coating, the PET can be optionally treated with oxygen plasma (50 w, 30 s, Femto Science FT12-EP078) before coating. To further improve the adhesion and mechanical duration, graphene oxide (GO) solution was optionally coated on the top surface of AgNWs. Without specifical description, the AgNWs surface was over-coated with a GO layer and post-annealed at 100 °C for 10 min followd by DI water wash for 2 times.

### Antenna measurements

Measurements used a meandered dipole antenna with dimensions of 126 mm × 15 mm, minimum size feature of 0.4 mm, and center frequency of 950 MHz. A 50 Ω sub-miniature version A (SMA) connector was soldered to a 2 mm × 2 mm copper foil and attaching to the connection pads of the antenna using conductive paste. S-parameters were measured using a coaxial cable (BU-4150029024, Mueller Electric Co) and a vector network analyzer (VNA, FieldFox N9915A). The radiation pattern was measured in a 5.5 m × 3.5 m × 3.5 m RF anechoic chamber lined with 0.5 m height pyramidal absorbing foam. The dipole antenna was placed on a rotary table (AL-4903-3A, 1° step size, and 10% rotation velocity) and a horn antenna (ANT-DR18S) was placed 4.5 m away. For all measurements, the input power was set to 10 dBm.

### RF tag sensor integration

Three RF tag sensors were fabricated with overall dimensions 93 mm × 1 mm (Fig. 1f window), 93 mm × 8 mm (Fig. 1f book), and 70 mm × 15 mm (Fig. 1f arm). The first two tag sensors use chip MR6-p. The last one uses Ucode8 (U8). All the tag sensors follow the half-wave dipole antenna design and possess similar size with the commercial one. The electrical connection between chip and tag sensor antennas is formed via using conductive paint (Ted Pella, Inc, Pelco 16062). To ensure a robust conductive connection between tag sensor and chip, the conductive paint was applied as thin as possible and was baked around 100 °C for 10 min. All the tag sensor antennas are designed for working under ultrahigh radio frequency around 860–960 MHz. For wireless temperature measurement, chip NMV2D CAB0 was used on tag sensor.

### RF tag sensor measurements

The measurement system includes tag sensor, reader, reader antenna, computer. The RFID system uses the EPCglobal standard. The reader (Impinj Speedway R420) was connected to computer via networking cable. The reader antenna was secured on a tripod and connected to the reader with a coaxial cable. The tag sensor was placed in the front of the reader antenna, and the Received Signal Strength Indicator (RSSI) value was recorded from software (ItemTest 2.5). According to Federal Communications Commission (FCC) regulations for electronic devices in the UHF RFID band, the maximum effective isotropic radiated power (EIRP) allowed is 36 dBm. Experiments involved varying the reading distance, reading angle, the deformation of the tag sensor, and performance on moving objects and human subjects. For wireless temperature measurements, a UHF-R200 reader was used.

### Optical and electrical characterization

The morphologies of AgNWs films and patterns were measured with Field-emission Scanning Electron Microscopy (FESEM) (FEI Verios460) and Optical Microscope. Transmittance measurement was conducted with UV-vis Spectrophotometer (Shimadze UV-1800) against a blank PET substrate. Resistance was measured with multimeter (2709B, BK Precision). Two silver paste electrodes were prepared on test sample first to reduce the contact resistance between measuring probe of multimeter and AgNWs.

## Supplementary Material

Supporting information

Movie S1

Movie S2

Movie S3

Movie S4

## Data Availability

All data supporting the findings of this study are available within the article and its Supplementary Information. Additional raw data generated in this work are available from the corresponding author upon reasonable request.

## References

[1] Haque A, Milstein A & Li F Illuminating the dark spaces of healthcare with ambient intelligence. Nature 585, 193–202 (2020). 32908264 10.1038/s41586-020-2669-y

[2] Acampora G, Cook DJ, Rashidi P & Vasilakos AV A Survey on ambient intelligence in healthcare. Proc. IEEE 101, 2470–2494 (2013). 10.1109/JPROC.2013.2262913 PMC3890262 24431472

[3] Attal F. Physical human activity recognition using wearable sensors. Sens. 15, 31314–31338 (2015). 10.3390/s151229858 PMC4721778 26690450

[4] Sharma P, Hui X, Zhou J, Conroy TB & Kan EC Wearable radio-frequency sensing of respiratory rate, respiratory volume, and heart rate. npj Digit. 3, 98 (2020). 10.1038/s41746-020-0307-6 PMC7387475 32793811

[5] Shao Y. Room-temperature high-precision printing of flexible wireless electronics based on MXene inks. Nat. Commun 13, 3223 (2022). 35680851 10.1038/s41467-022-30648-2 PMC9184614

[6] Niu S. A wireless body area sensor network based on stretchable passive tag sensors. Nat. Electron 2, 361–368 (2019).

[7] Sarycheva A. Utilization of synergistic effect of dimension-differentiated hierarchical nanomaterials for transparent and flexible wireless communication. Sci. Adv 4, eaau0920 (2018).30255151

[8] Debes C. Monitoring activities of daily living in smart homes: understanding human behavior. IEEE Signal Process. Mag 33, 81–94 (2016).

[9] Usman M. Intelligent wireless walls for contactless in-home monitoring. Light Sci. Appl 11, 212 (2022). 35798702 10.1038/s41377-022-00906-5 PMC9262883

[10] Patel S, Park H, Bonato P, Chan L & Rodgers M A review of wearable sensors and systems with application in rehabilitation. J. Neuroeng. Rehabil 9, 21 (2012). 22520559 10.1186/1743-0003-9-21 PMC3354997

[11] Li W, Akhter Z, Vaseem M & Shamim A Optically transparent and flexible radio frequency electronics through printing technologies. Adv. Mater. Technol 7, 2101277 (2022).

[12] Yang Y, Li W, Salama KN & Shamim A Polarization insensitive and transparent frequency selective surface for dual band GSM shielding. IEEE Trans. Antennas Propag 69, 2779–2789 (2021).

[13] Goliya Y. Next generation antennas based on screen-printed and transparent silver nanowire films. Adv. Opt. Mater 7, 1900995 (2019).

[14] Hautcoeur J, Colombel F, Himdi M, Castel X & Cruz EM Large and optically transparent multilayer for broadband H-shaped slot antenna. IEEE Antennas Wirel. Propag. Lett 12, 933–936 (2013).

[15] Green RB Optically transparent antennas and filters. IEEE Antennas Propag. 61, 37–47 (2019).

[16] Zhang Y. High precision epidermal radio frequency antenna via nanofiber network for wireless stretchable multifunction electronics. Nat. Commun 11, 5629 (2020). 33159080 10.1038/s41467-020-19367-8 PMC7648760

[17] Kim J. Wearable smart sensor systems integrated on soft contact lenses for wireless ocular diagnostics. Nat. Commun 8, 14997 (2017). 28447604 10.1038/ncomms14997 PMC5414034

[18] Kim J. A soft and transparent contact lens for the wireless quantitative monitoring of intraocular pressure. Nat. Biomed. Eng 10, 1–11 (2021). 10.1038/s41551-021-00719-8 33941897

[19] Zhang Z. Stretchable transparent wireless charging coil fabricated by negative transfer printing. ACS Appl. Mater. Interfaces 11, 40677–40684 (2019). 31589402 10.1021/acsami.9b14728

[20] Wan T. Facile patterning of silver nanowires with controlled polarities via inkjet-assisted manipulation of interface adhesion. ACS Appl. Mater. Interfaces 12, 34086–34094 (2020). 32643927 10.1021/acsami.0c07950

[21] Baumbauer CL Printed, flexible, compact UHF-RFID sensor tag sensors enabled by hybrid electronics. Sci. Rep 10, 16543 (2020). 33024141 10.1038/s41598-020-73471-9 PMC7538943

[22] Wang Y. Flexible RFID tag sensor metal antenna on paper-based substrate by inkjet printing technology. Adv. Funct. Mater 29, 1902579 (2019).

[23] Wang H. High-performance transparent broadband microwave absorbers. Adv. Mater. Interfaces 9, 2101714 (2022).

[24] Ahn SH & Guo LJ High-speed roll-to-roll nanoimprint lithography on flexible plastic substrates. Adv. Mater 20, 2044–2049 (2008).

[25] Lu X, Zhang Y & Zheng Z Metal-based flexible transparent electrodes: challenges and recent advances. Adv. Electron. Mater 7, 2001121 (2021).

[26] Lagrange M. Optimization of silver nanowire-based transparent electrodes: effects of density, size and thermal annealing. Nanoscale 7, 17410–17423 (2015). 26437607 10.1039/c5nr04084a

[27] Jang H, Kim D, Tak H, Nam J & Kim T Ultra-mechanically stable and transparent conductive electrodes using transferred grid of Ag nanowires on flexible substrate. Curr. Appl. Phys 16, 24–30 (2016).

[28] Langley D. Flexible transparent conductive materials based on silver nanowire networks: a review. Nanotechnology 24, 452001 (2013). 24121527 10.1088/0957-4484/24/45/452001

[29] He W & Ye C Flexible transparent conductive films on the basis of Ag nanowires: design and applications: a review. J. Mater. Sci. Technol 31, 581–588 (2015).

[30] Jin Y, Deng D, Cheng Y, Kong L & Xiao F Annealing-free and strongly adhesive silver nanowire networks with long-term reliability by introduction of a nonconductive and biocompatible polymer binder. Nanoscale 6, 4812–4818 (2014). 24664157 10.1039/c3nr05820d

[31] Finn DJ, Lotya M & Coleman JN Inkjet printing of silver nanowire networks. ACS Appl. Mater. Interfaces 7, 9254–9261 (2015). 25874531 10.1021/acsami.5b01875

[32] Wu X, Zhou Z, Wang Y & Li J Syntheses of silver nanowires ink and printable flexible transparent conductive film: a review. Coatings 10, 865 (2020).

[33] Pan K. Sustainable production of highly conductive multilayer graphene ink for wireless connectivity and IoT applications. Nat. Commun 9, 5197 (2018). 30518870 10.1038/s41467-018-07632-w PMC6281590

[34] Yang S, Vaseem M & Shamim A Fully inkjet-printed VO_2_-based radio-frequency switches for flexible reconfigurable components. Adv. Mater. Technol 4, 1800276 (2019).

[35] Nair NM Printable silver nanowire and PEDOT:PSS nanocomposite ink for flexible transparent conducting applications. ACS Appl. Electron. Mater 2, 1000–1010 (2020).

[36] Li W, Yang S & Shamim A Screen printing of silver nanowires: balancing conductivity with transparency while maintaining flexibility and stretchability. Npj Flex. Electron 3, 13 (2019).

[37] Park K. High-resolution and large-area patterning of highly conductive silver nanowire electrodes by reverse offset printing and intense pulsed light irradiation. ACS Appl. Mater. Interfaces 11, 14882–14891 (2019). 30919616 10.1021/acsami.9b00838

[38] Li W, Yarali E, Bakytbekov A, Anthopoulos TD & Shamim A Highly transparent and conductive electrodes enabled by scalable printing-and-sintering of silver nanowires. Nanotechnology 31, 395201 (2020). 32531776 10.1088/1361-6528/ab9c53

[39] Liang C. Surface ablation thresholds of femtosecond laser micropatterning silver nanowires network on flexible substrate. Microelectron. Eng 232, 111396 (2020).

[40] Hwang JS, Park JE, Kim GW, Lee H & Yang M Near-infrared nanosecond pulsed laser ablation of silver nanowire in aqueous media for low-power and low-debris laser processing. J. Micromech. Microeng 30, 115014 (2020).

[41] Sopeña P, Serra P & Fernández-Pradas JM Transparent and conductive silver nanowires networks printed by laser-induced forward transfer. Appl. Surf. Sci 476, 828–833 (2019).

[42] Cann M. High performance transparent multi-touch sensors based on silver nanowires. Mater. Today Commun 7, 42–50 (2016).

[43] Sarycheva A. 2D titanium carbide (MXene) for wireless communication. Sci. Adv 4, eaau0920 (2018). 30255151 10.1126/sciadv.aau0920 PMC6155117

[44] Park SE, Kim S, Lee DY, Kim E & Hwang J Fabrication of silver nanowire transparent electrodes using electrohydrodynamic spray deposition for flexible organic solar cells. J. Mater. Chem. A 1, 14286–14293 (2013).

[45] Marques AHF Environmentally friendly, semi-transparent, screen printed antenna for RFID tag applications. Braz. J. Phys 51, 434–438 (2021).

[46] Liang J. Silver nanowire percolation network soldered with graphene oxide at room temperature and its application for fully stretchable polymer light-emitting diodes. ACS Nano. 8, 1590–1600 (2014). 24471886 10.1021/nn405887k

[47] Tag sensor performance parameters and test methods v1.1.3. electronic product code (EPC) global standard, Brussels, Belgium (2005).

[48] Son HW & Jeong SH Wideband RFID tag sensor antenna for metallic surfaces using proximity-coupled feed. IEEE Antennas Wirel. Propag. Lett 10, 377–380 (2011).

[49] FCC electronic code of federal regulations. https://www.ecfr.gov/cgi-bin/text-idx?SID=eed706a2c49fd9271106c3228b061%5f3&mc=true&node=pt47.1.15&rgn=div5 (2020).

[50] Tajin MAS, Mongan WM & Dandekar KR Passive RFID-based diaper moisture sensor. IEEE Sens. J 21, 1665–1674 (2021).

[51] Lin R. Wireless battery-free body sensor networks using near-field-enabled clothing. Nat. Commun 11, 444 (2020). 31974376 10.1038/s41467-020-14311-2 PMC6978350

[52] Beauchet O. Gait variability among healthy adults: low and high stride-to-stride variability are both a reflection of gait stability. Gerontology 55, 702–706 (2009). 19713694 10.1159/000235905

[53] Zhang J, Tian G, Marindra A, Sunny A & Zhao A A review of passive RFID tag sensor antenna-based sensors and systems for structural health monitoring applications. Sensors 17, 265 (2017). 28146067 10.3390/s17020265 PMC5335955

[54] Abuelkhail A, Baroudi U, Raad M & Sheltami T Internet of things for healthcare monitoring applications based on RFID clustering scheme. Wirel. Netw 27, 747–763 (2021).

[55] Ho L, Moh M, Walker Z, Hamada T & Su CF A prototype on RFID and sensor networks for elder healthcare: progress report. Proc. 2005 ACM Sigcomm. Work Exp. Approaches Wirel. Netw. Des. Anal. - E-wind 05, 70–75 (2005).

[56] Tsirmpas C, Rompas A, Fokou O & Koutsouris D An indoor navigation system for visually impaired and elderly people based on Radio Frequency Identification (RFID). Inf 320, 288–305 (2015).

[57] Oguntala GA SmartWall: Novel RFID-enabled ambient human activity recognition using machine learning for unobtrusive health monitoring. IEEE Access 7, 68022–68033 (2019).

[58] Hui X & Kan EC Monitoring vital signs over multiplexed radio by near-field coherent sensing. Nat. Electron 1, 74–78 (2018).

[59] Liu YC Monitoring gait at home with radio waves in Parkinson’s disease: A marker of severity, progression, and medication response. Sci. Transl. Med 14, eadc9669 (2022). 36130014 10.1126/scitranslmed.adc9669 PMC12853226

[60] Yang YZ Artificial intelligence-enabled detection and assessment of Parkinson’s disease using nocturnal breathing signals. Nat. Med 28, 2207–2215 (2022). 35995955 10.1038/s41591-022-01932-x PMC9556299

[61] Rivadeneyra A. Laser-Fabricated Antennas for RFID Applications. 2020 50th Eur. Microw. Conf. Eumc. 00, 812–815 (2021).

[62] Park JK Remotely triggered assembly of 3D mesostructures through shape-memory effects. Adv. Mater 31, 1905715 (2019). 10.1002/adma.201905715 31721341

[63] Ma Y. Flexible hybrid electronics for digital healthcare. Adv. Mater 32, 1902062 (2020). 10.1002/adma.201902062 31243834

[64] Yang S. “Cut-and-Paste” Manufacture of multiparametric epidermal sensor systems. Adv. Mater 27, 6423–6430 (2015). 26398335 10.1002/adma.201502386

[65] Yang Y. A laser-engraved wearable sensor for sensitive detection of uric acid and tyrosine in sweat. Nat. Biotechnol 38, 217–224 (2019). 31768044 10.1038/s41587-019-0321-x

[66] Hofmann O, Stollenwerk J & Loosen P Design of multi-beam optics for high throughput parallel processing. J. Laser Appl 32, 012005 (2020).

[67] Hong N. Roll-to-roll dry transfer of large-scale graphene. Adv. Mater 34, 2106615 (2022). 10.1002/adma.202106615 34751484

[68] Crawford THR, Borowiec A & Haugen HK Femtosecond laser micro-machining of grooves in silicon with 800 nm pulses. Appl. Phys 80, 1717–1724 (2005).

[69] Cao Q & Rogers JA Ultrathin films of single-walled carbon nanotubes for electronics and sensors: a review of fundamental and applied aspects. Adv. Mater 21, 29–53 (2009).

[70] Layani M, Kamyshny A & Magdassi S Transparent conductors composed of nanomaterials. Nanoscale 6, 5581–5591 (2014). 24777332 10.1039/c4nr00102h

