# Supplementary material for: Digitally-defined ultrathin transparent wireless sensor network for room-scale imperceptible ambient intelligence
Source: Npj Flex Electron. Author manuscript; Available in PMC 2024 Dec 5. (PMC11619817; doi:10.1038/s41528-024-00293-4)
Supplement: Supporting information [file NIHMS1982860-supplement-Supporting_information.docx]

Supporting information

**Digitally-defined ultrathin transparent wireless sensor network for room-scale imperceptible ambient intelligence**

*Yunxia Jin^1,6^, Mengxia Yu^2^, Dat T. Nguyen^3^, Xin Yang^2^, Zhipeng Li^2^, Ze Xiong^1^, Chenhui Li^2^, Yuxin Liu^1,4,6,7^, Yong Lin Kong^5*^, John S. Ho^1,2,4*^*

*Corresponding author. ^*^E-mail: yong.kong@utah.edu, johnho@nus.edu.sg


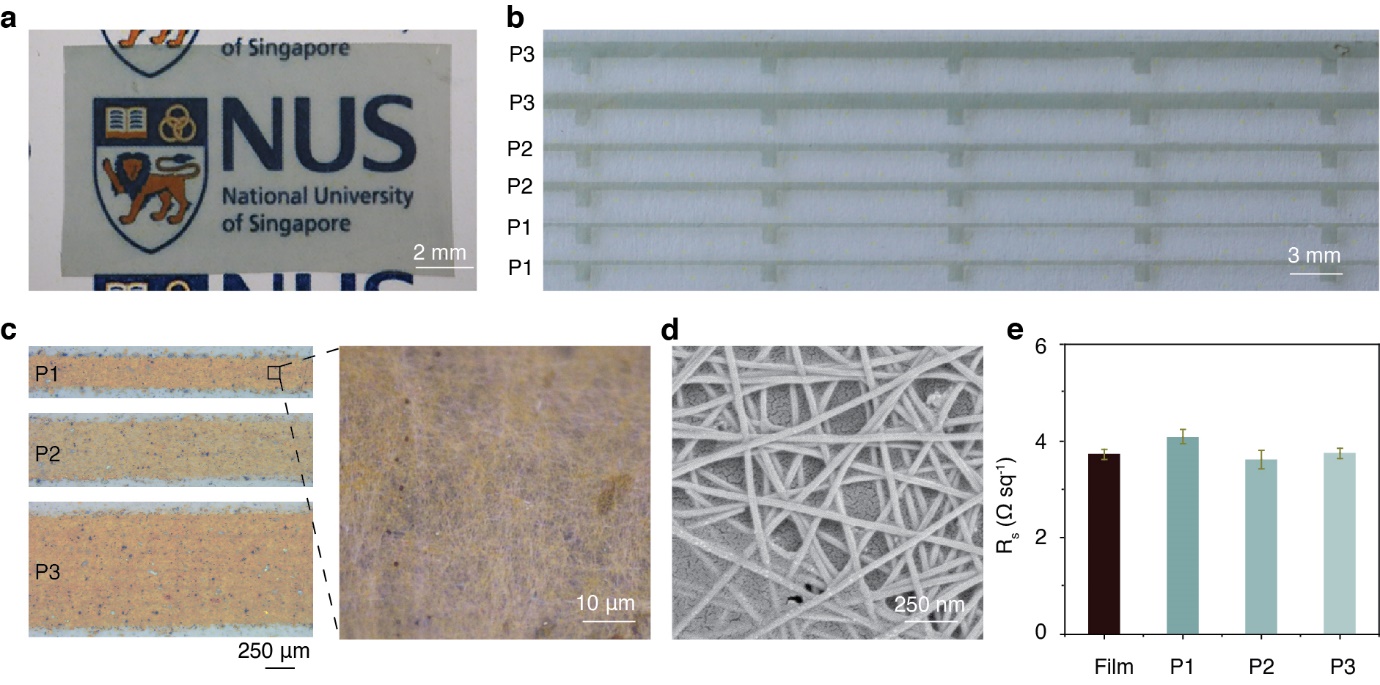


**Supplementary Figure 1.** **Conductivity of fabricated traces.** **a** Photograph of part of the transparent conductive AgNW film before patterning. **b** Photograph of traces in varying widths fabricated using our method. **c** Optical images of the traces. Right optical image is the Zoom in from left images. **d** SEM image of the traces. The bottom left side shows GO covering on the top. **e** Comparison of sheet resistance between pristine film and the patterned traces with different widths. Error bar is the standard deviation of sheet resistance.


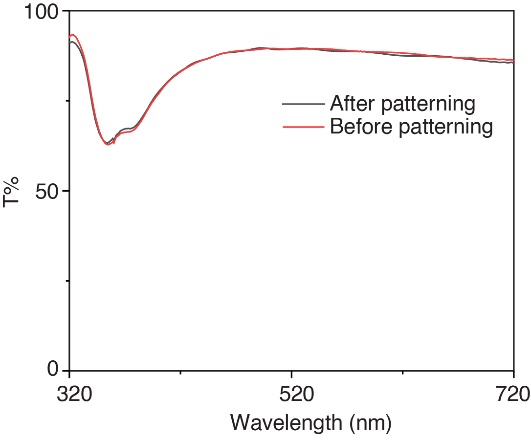


**Supplementary Figure 2.** **Transmittance of the transparent sensor before and after patterning.** They show almost the same transmittance, indicating no transparency change due to patterning process.

Supplementary Table 1 Patterning methods comparison.

Remarks: Typical printing, photolithography, laser-based methods are compared regarding the maximum demo area of patterning, resolution of the device demonstrated, opto-electrical performance and RF performance. The Printing methods either result in panel-designed patterns with low resolution or trace-designed patterns with higher resolution but at a cost of reduced conductivity resulting from the addition of insulating polymer to adjust printability. There is no report of transparent RF device with high RF performance from printing methods. Photolithography can reach high resolution at wafer scale, but it involves mask fabrication, photoresist usage, etching, washing etc., which is complex and high-cost, so it is not suitable for large-scale and low-cost RF devices fabrication. Laser ablation is able to reach high resolution as well especially using nano-pulsed laser cutter, but its serial spot-by spot etching makes it at very low productivity, so it is time-consuming and subsequently high-cost for RF devices. The method developed here combines low-cost and rapid laser cutting with adhesion-controlled peeling to fabricate transparent AgNWs patterns. A water-soluble tape was cut first without penetrating transparent film behind, and it can peel off the unwanted AgNWs part completely in a single pass in air, while detaching completely from the wanted AgNWs part in water less than 3 min. No chemical, etching, or high-energy post-treatment are required. Therefore, it is low-cost, scalable and highly reproducible.


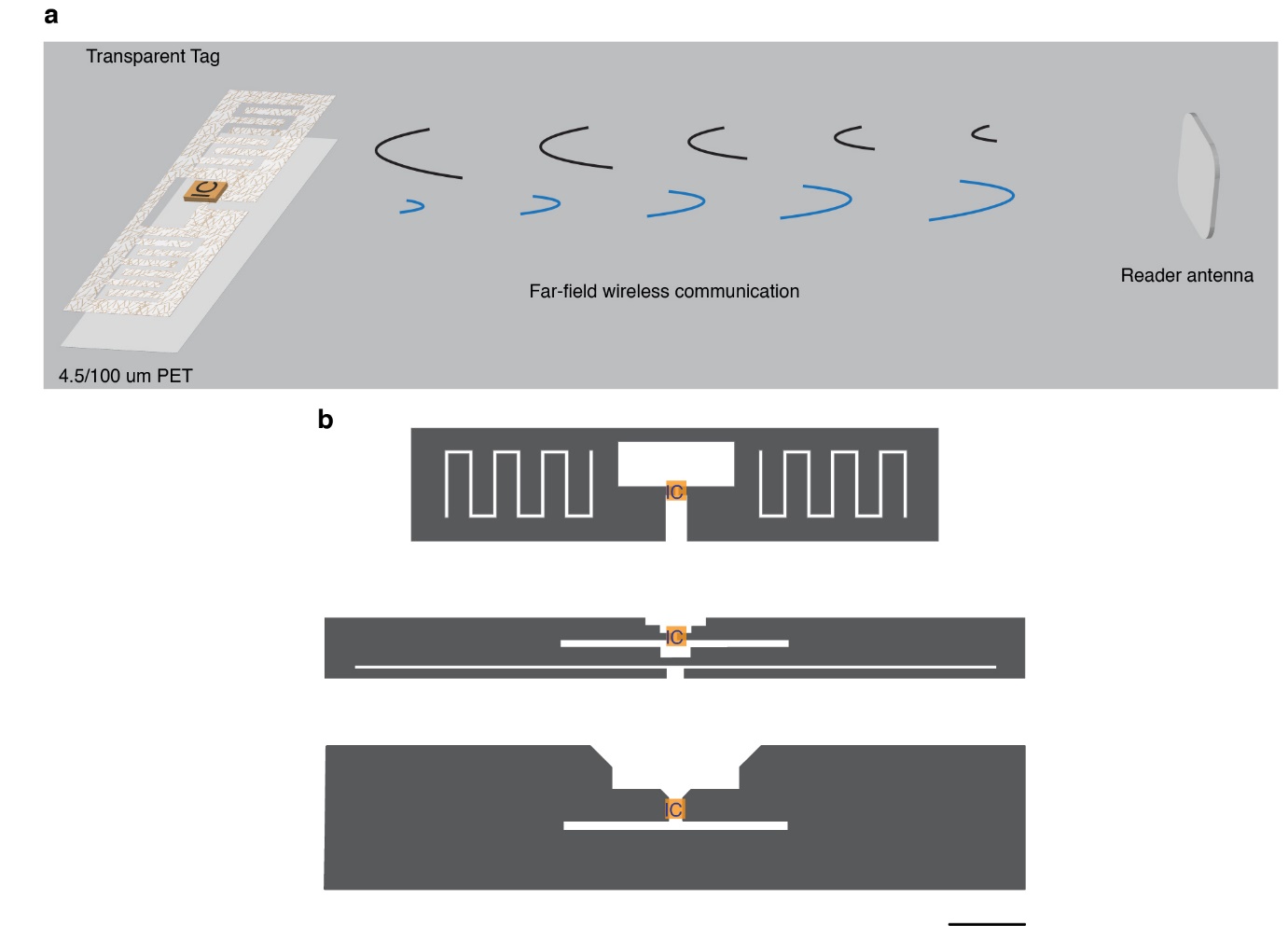


**Supplementary Figure 3.** **RF system and tag design.** **a** Schematic of the RF system. The system is based on a UHF RFID Reader (840-960 MHz) with a maximum transmit power of 32.5 dBm and receive sensitivity of -84 dBm. **b** Schematics of the tag designs: meandered tag, slot tag and bulk tag. The tags are based on dipole antennas of varying dimensions and gain. Scale bar is 1 cm.


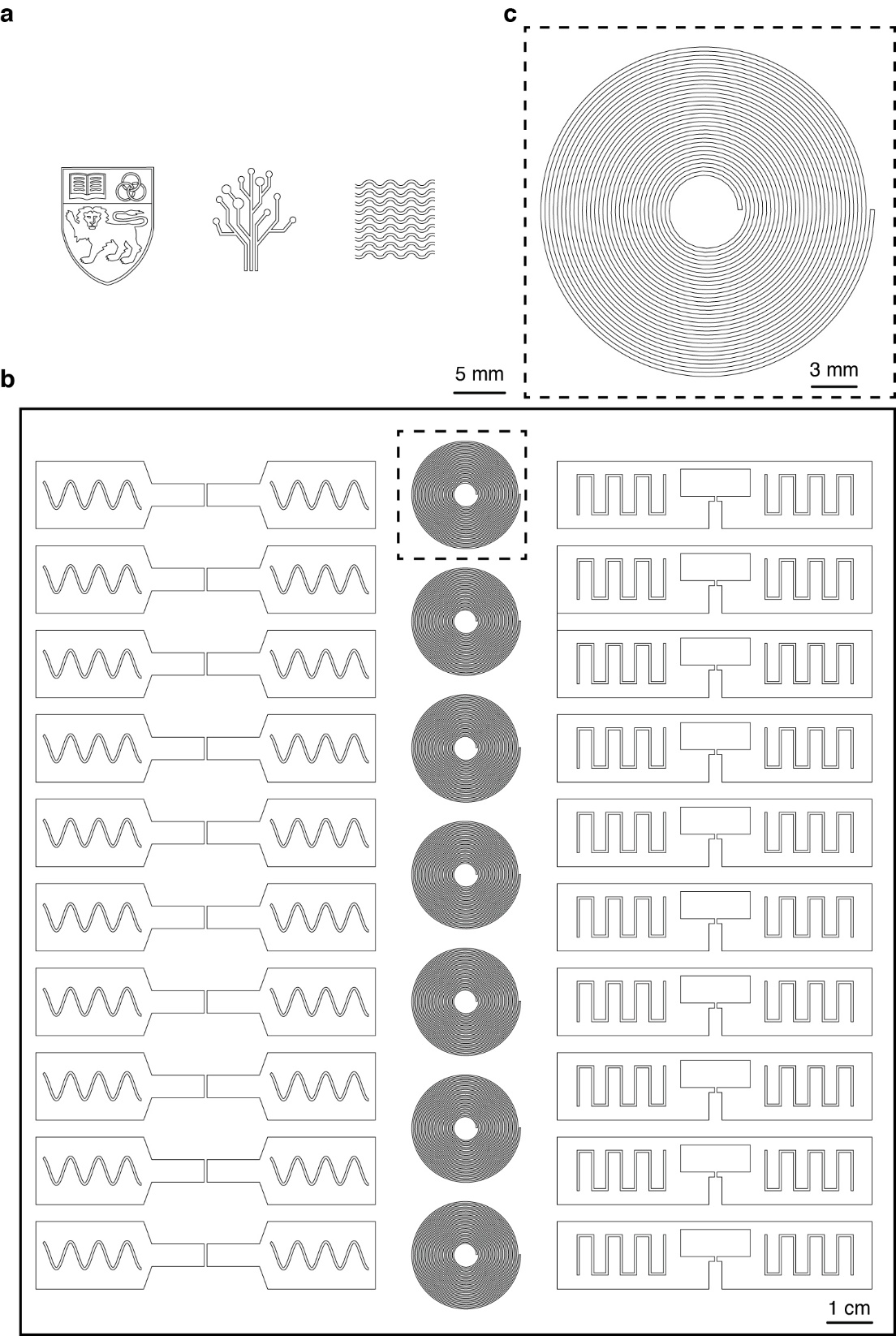


**Supplementary Figure 4. Drawings of the fabrication patterns.** **a** Drawing of the patterns shown in **Fig. 2b**. **b** Drawing of the sheet of 27 tags shown in **Fig. 2a**. **c** Zoom in of the inductor pattern in the dotted black box in **b**.


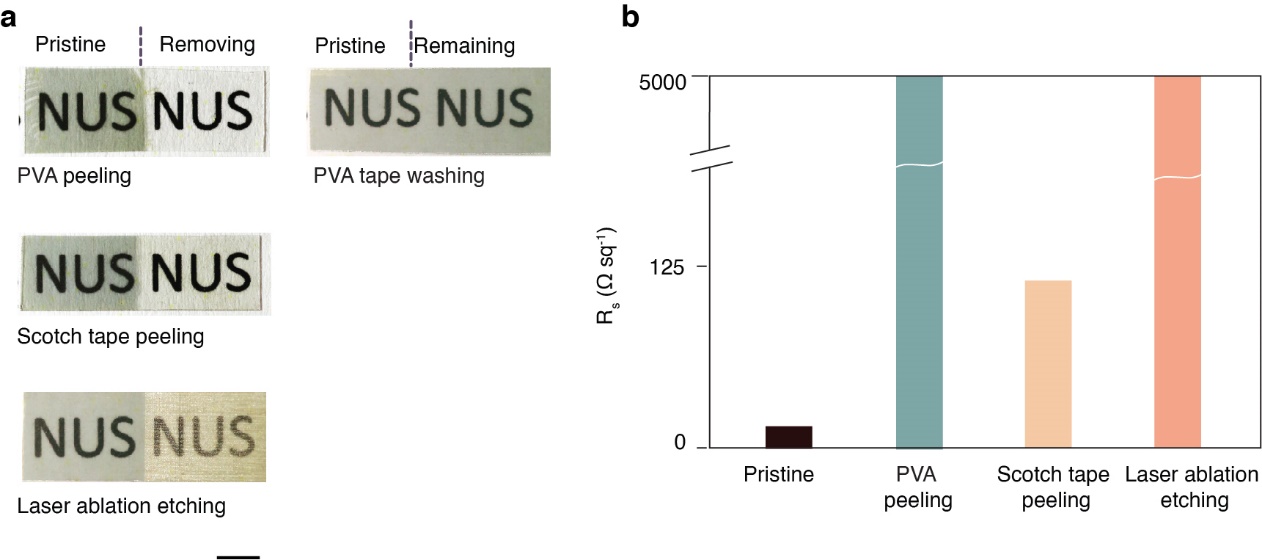


**Supplementary Figure 5.** **Comparison of patterning methods.** **a** Photographs of AgNWs on a thin and transparent substrate in pristine state (left) and after attempting to remove the AgNWs by peeling with PVA tape, peeling with Scotch tape, and laser ablation (right). The background shows the letters “NUS” for visual comparison. The photograph on the top right shows AgNWs in pristine state and after PVA tape has been applied and washed away using water without peeling. **b** Sheet resistance of the samples in **a**.


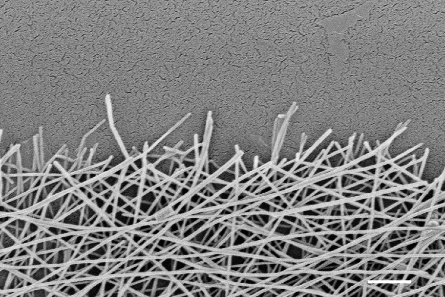


**Supplementary Figure 6. SEM image of the AgNWs film after removing part AgNWs by peeling with PVA tape.** It shows clear AgNWs along the edge where one side is completely removed while the other side remains intact. The scale bar is 500 nm.


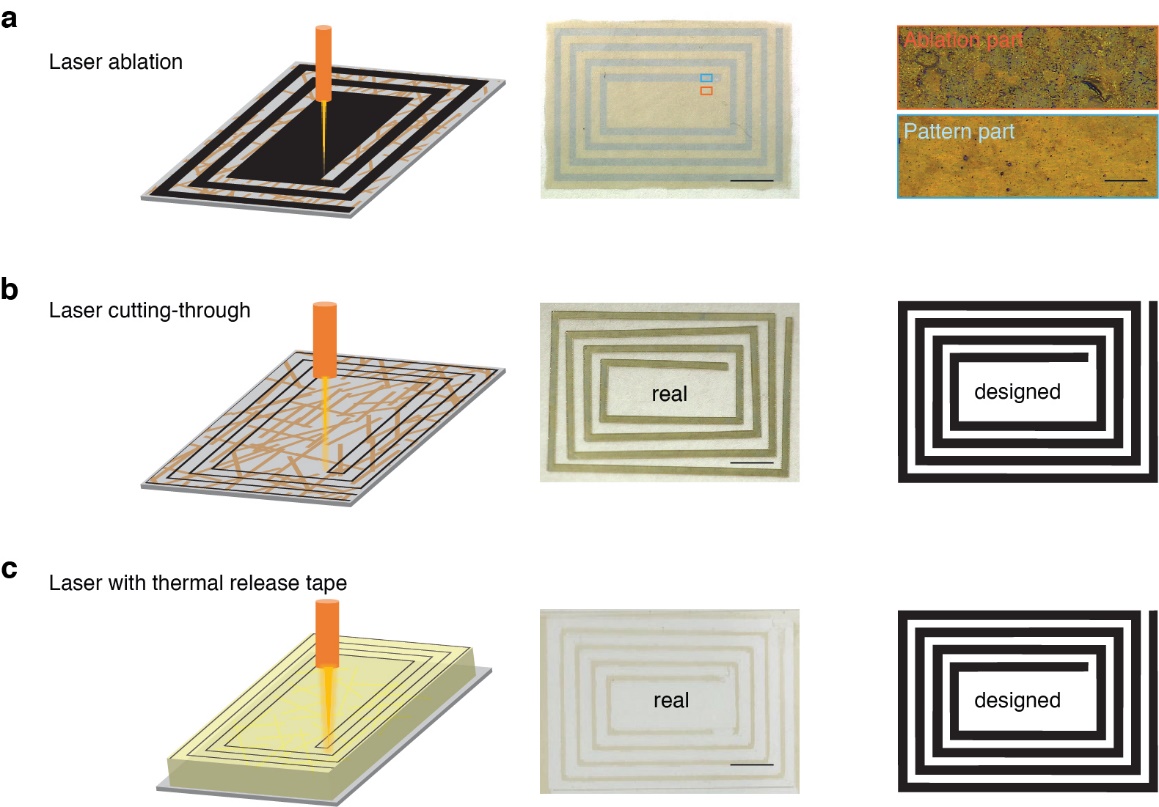


**Supplementary Figure 7.** **Comparison of laser patterning.** **a** Schematic of laser ablation, photograph of AgNWs pattern fabricated by laser ablation, and optical image of the ablation part and pattern part. It induces nanodroplets of silver among patterns as shown in optical image here and SEM image in Figure 2c. **b** Schematic of laser cutting-through, photograph of AgNWs pattern after laser cutting, and the drawing of the pattern design used by the laser cutter. It causes a deformed shape of coils compared to the pre-designed pattern in the right side as the free-moving of cutting parts. **c** Schematic of Laser blind cutting with thermal release tape, photograph of AgNWs pattern, and the drawing of the pattern design. It results in a pattern with much narrower width compared with the design due to the higher laser cutting energy for thicker release tape and incomplete adhesion release of the tape after thermal heating. Scale bar in the center column is 5 mm. Scale bar in the right top is 0.2 mm.


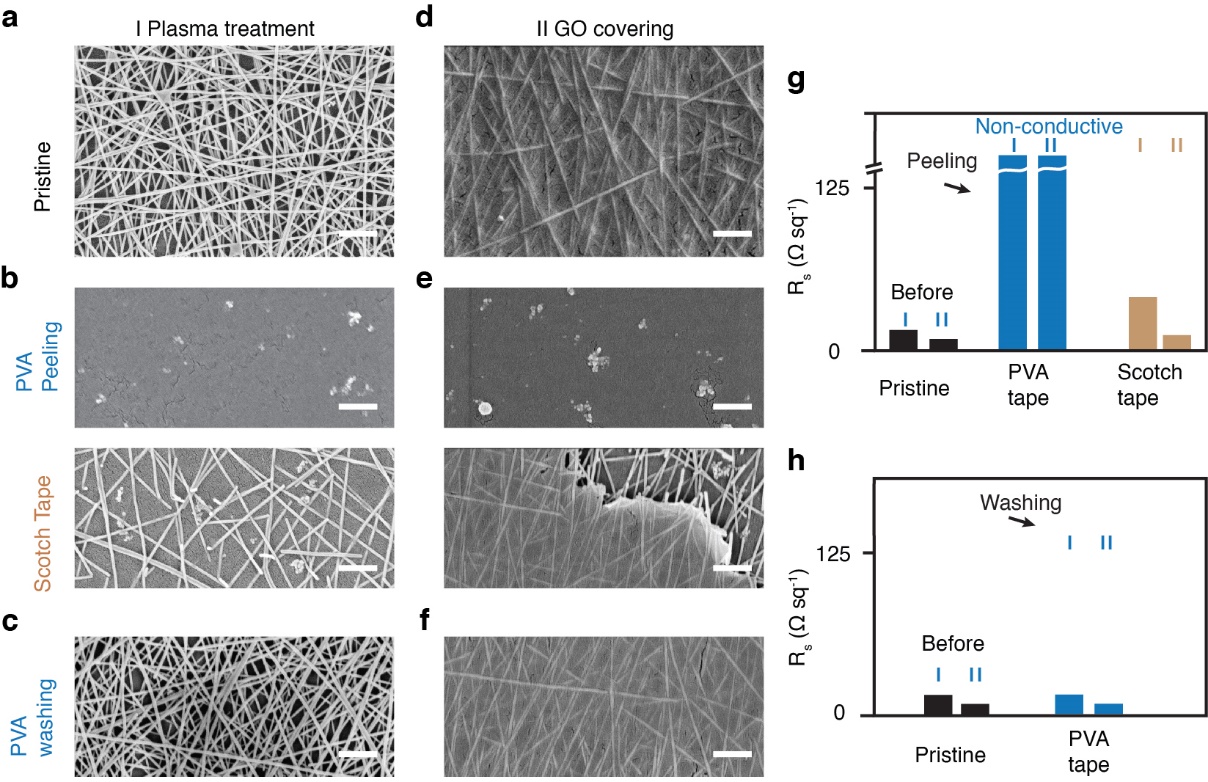


**Supplementary Figure 8.** **Patterning of GO-treated AgNW film.** **a-c** SEM image of AgNWs on plasma-treated PET substrate in the pristine state (**a**), after peeling using PVA tape (top) and Scotch tape (bottom) (**b**), and after applying and washing PVA tape without peeling using water (**c**). **d-f** SEM images of AgNWs film coated with graphene oxide (GO) in the pristine state (**d**), after peeling using PVA tape (top) and Scotch tape (bottom) (**e**), and and after applying and washing PVA tape without peeling (**f**). **g** Sheet resistance of the AgNWs film with plasma treatment and GO coating in the pristine state and after peeling using PVA tape and Scotch tape. **h** Sheet resistance before and after washing each of the samples. All scale bars are 500 nm. With plasma and GO over-coating, the PVA tape is still capable of removing the AgNWs in a single round, while the regular Scotch tape leave more AgNWs on the substrate after peeling compared to non-treated AgNWs networks, and almost lose the peeling capability after GO over-coating. There are some nanoscale dots shown on SEM images after PVA tape peeling, which are the sticking leftover of AgNWs due to enhanced adhesion among AgNWs, substrate and GO. But they are visually invisible and cause the film to lose conductivity.


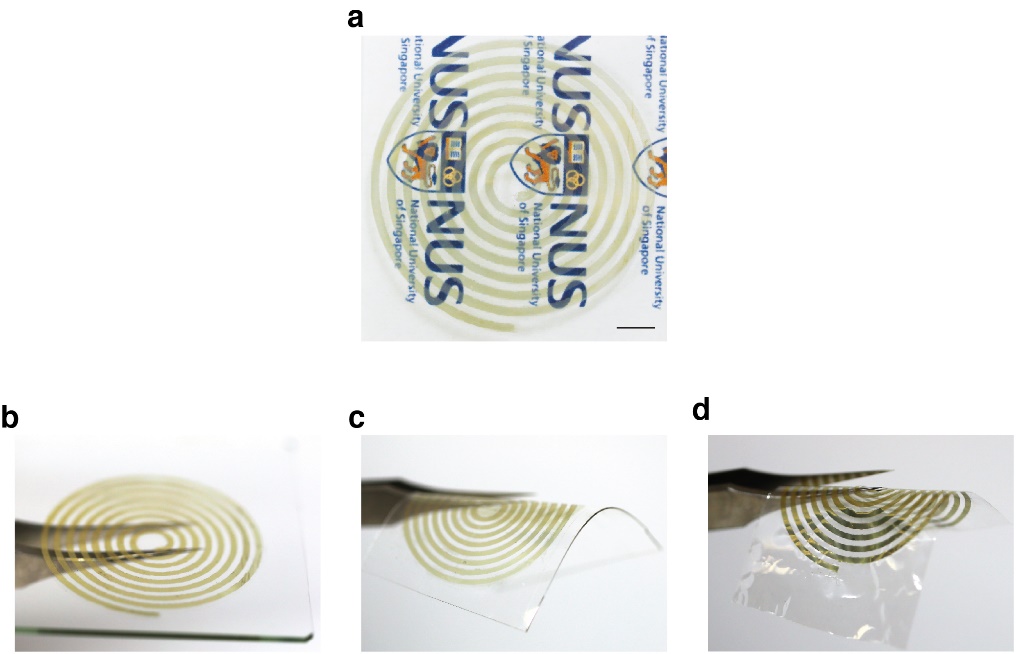


**Supplementary Figure 9.** **Patterning on rigid and flexible substrates.** **a-d** Inductor fabricated from transparent conductive AgNWs film at 71.4% transmittance and 6.5 Ω sq^-1^ sheet resistance against a letter background (**a**), on glass (**b**), PDMS (**c**), and 4.5-μm thick PET (**d**). The scale bar is 0.5 cm.


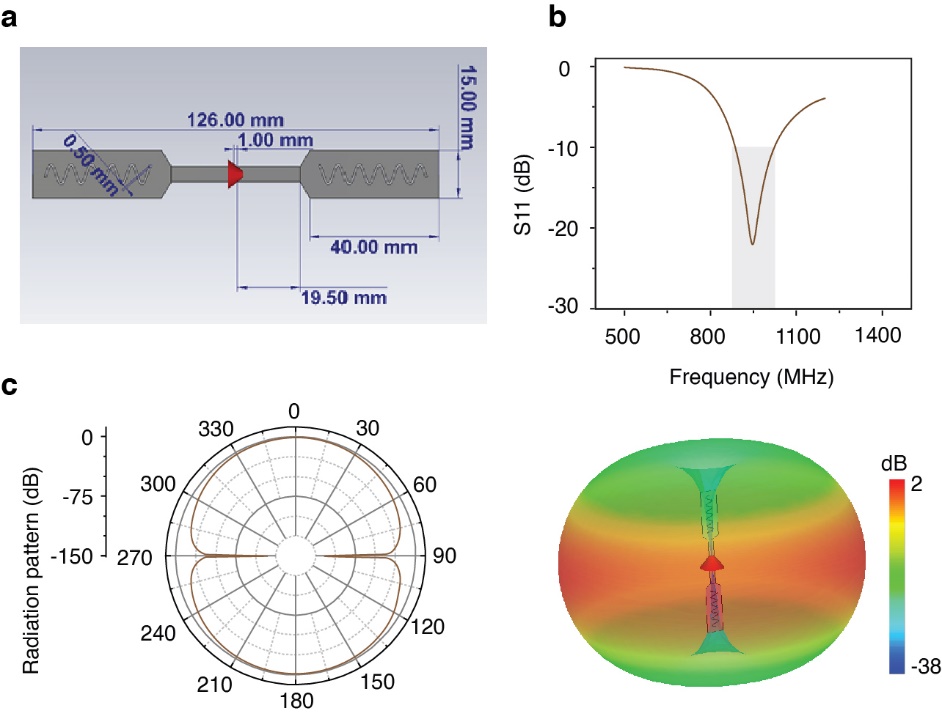


**Supplementary Figure 10.** **Antenna design and simulations. a** Design and dimensions of the dipole antenna. **b** Simulated ideal S11 spectrum of the dipole antenna made from perfect electric conductor (PEC). **c** Simulated radiation patterns in the E-plane (left) and in 3D (right).


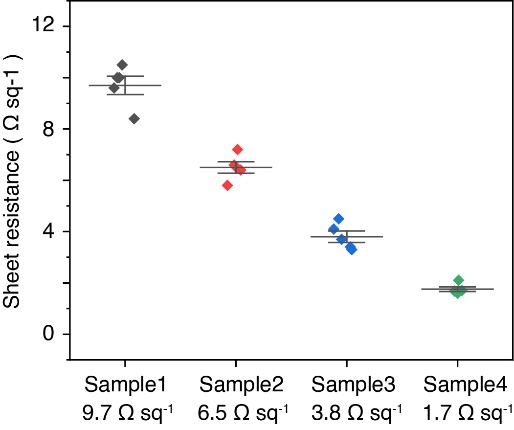


**Supplementary Figure 11. Sheet resistances of devices at different transmittance.** To ensure the measurement accuracy, we measured 5 locations on each sample.


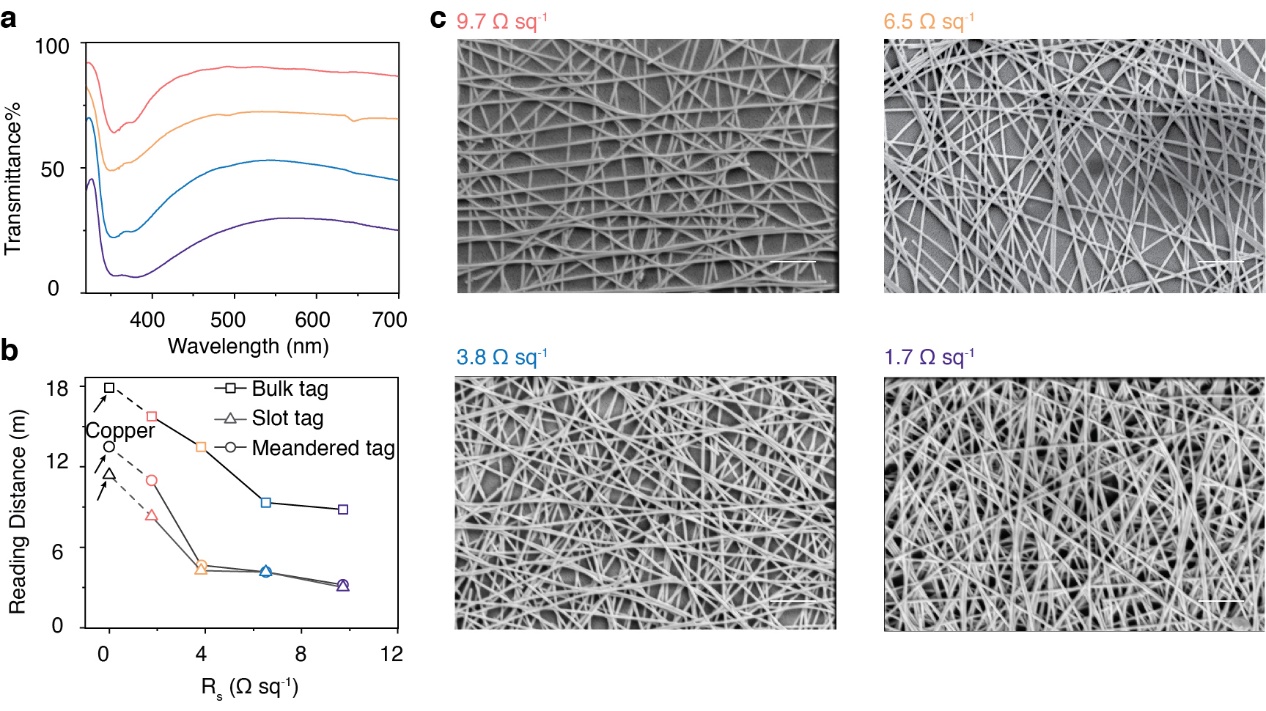


**Supplementary Figure 12.** **Transparent RFID sensing tag working range performance. a** UV-Vis spectra of transparent tags. **b** Reading range of copper-based and transparent tags. The first black dots are copper tag with the same pattern design as the transparent bulk tag, slot tag and meandered tag, respectively. Bulk tag has a reading distance of 8.0 m at 9.7 Ω sq^-1^, and extend to 15.2 m at 1.7 Ω sq-1, at which the tag still has clear visual transparency to look through object behind the tag. It is 84.4% of the same tag design made of copper. The slot tag has reading distance of 3.0 m and 8.0 m, while meandered tag has 3.0 m and 10.6 m at 9.7 and 1.7 Ω sq^-1^, respectively, which is corresponding to 71.2 and 81.5% of the copper tag. **c** SEM images of transparent tags with different sheet resistance. Higher transparency and lower sheet resistance shows sparse AgNWs deposition. The scale bar is 500 nm.


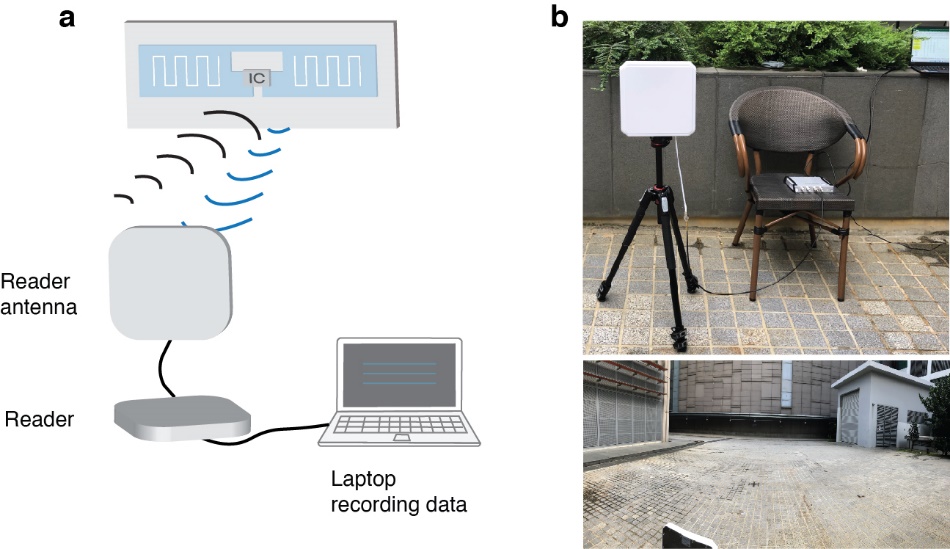


**Supplementary Figure 13.** **Experimental setup for RF range measurements. a** Schematic of the experimental setup. The antenna has a gain of 6 dBi and a transmit power of 30 dBm. **b** Photographs of the setup. For repeatability, the setup is placed in an open outdoor environment, except for the measurements in Fig. 4c-i and Fig. 5 which is placed in an indoor environment to be representative of ambient sensing applications.


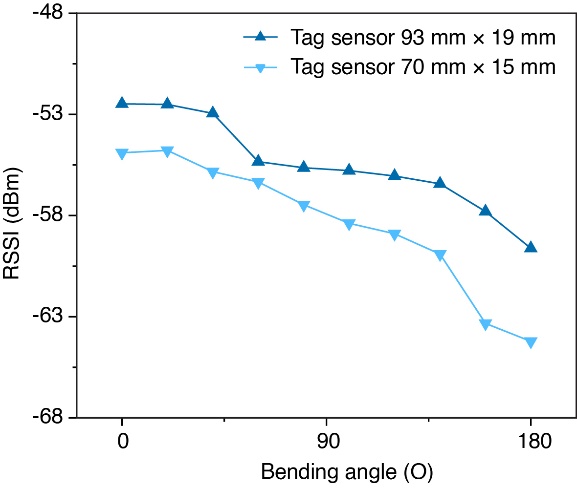


**Supplementary Figure 14. Tag sensor under bending**. RSSI change of transparent tag sensor under bending. The tags are still functional under bending. The higher the bending angle, the lower the RSSI.


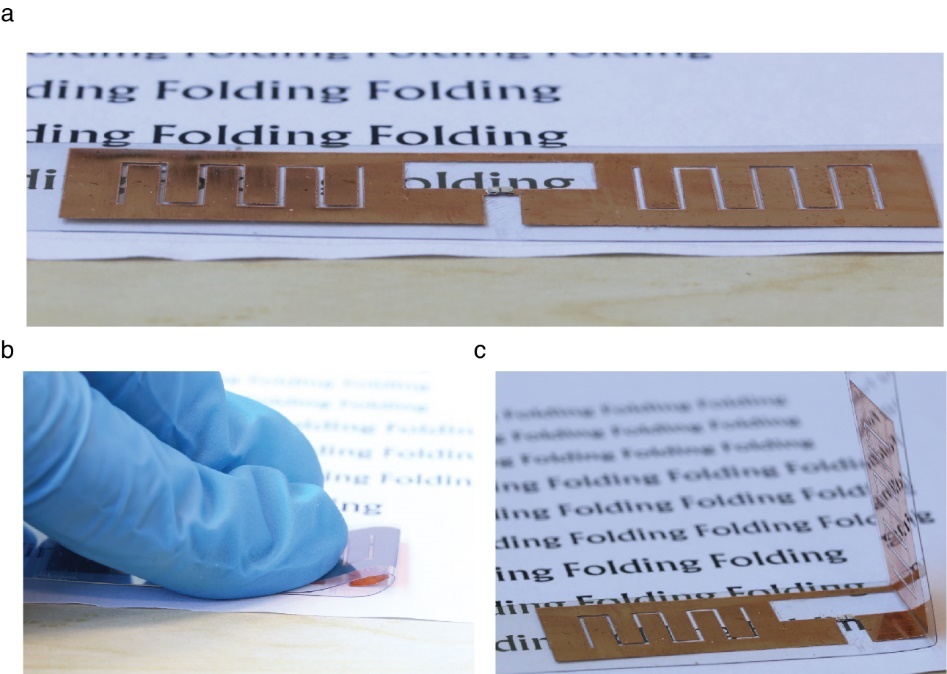


**Supplementary Figure 15. Comparison with a non-transparent copper RF tag. a** Photograph of a non-transparent RF tag placed on a flat surface with a text background. The tag consists of copper on a 100-μm thick polyethylene terephthalate (PET) substrate. **b** Tag in a folded state. **c** Tag after release from folding showing non-elastic deformation.


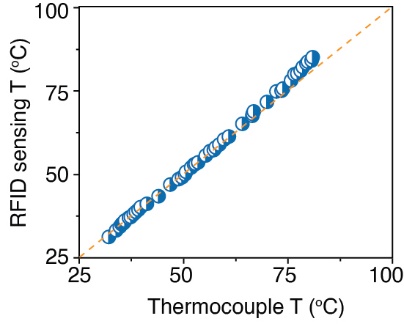


**Supplementary Figure 16.** **Temperature sensor calibration.** Temperature measured by the sensor on the RF tag as a function of temperature measured by a thermocouple. The dashed line is the fitting curve, which has a slope of 1.07. It suggests the temperature measured by RFID tag is almost the same as that by thermocouple.


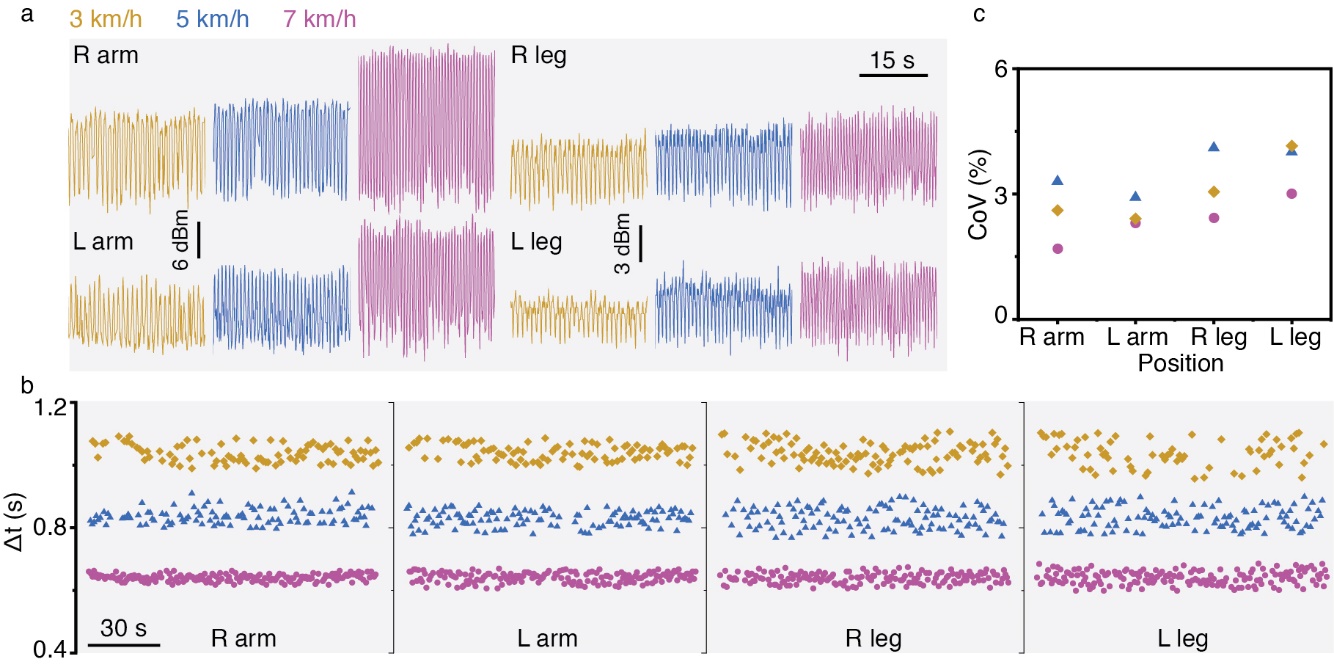


**Supplementary Figure 17.** **Ambient wireless sensing during exercise. a** RSSI trace recorded by the reader from RF tags placed on the arms and legs of a subject running on a treadmill at speeds 3, 5 and 7 km/h. The tags are placed between two layers of Tegaderm on the skin. The reader is placed in the front of the treadmill facing the subject. **b** Stride time Δt calculated from the peak-to-peak interval of the RSSI traces. **c** Stride time variability (STV) of all legs and arms. The STV is below 4% for all the positions, which is indicative of a healthy subject.

**Supplementary Movie 1**

Operating range of the transparent tag sensors using a reader antenna with gain of 6 dBi and maximum transmit power of 30 dBm.

**Supplementary Movie 2**

1000 cycles of folding and crumpling of transparent tag sensors. The tag sensors are placed 2 m from a reader antenna with a gain of 6 dBi and a maximum transmit power of 30 dBm.

**Supplementary Movie 3**

Multiplexed activity tracking from various tagging objects with transparent sensors.

**Supplementary Movie 4**

Motion tracking from 4 tag sensors placed on the arms and legs of a subject jogging on a treadmill at a speed of 3, 5 and 7 km h^-1^.
